# SNP234, a novel potent, aggregate selective anti‐Aβ antibody, as next generation anti‐amyloid immunotherapy amicable for subcutaneous administration

**DOI:** 10.1002/alz70859_107093

**Published:** 2025-12-26

**Authors:** Yuka A Martens, Jakob Samsel, Mary Elizabeth Curtis, Young Park, Zusheng Li, Kevin Chen, Gang An, Min Li

**Affiliations:** ^1^ SciNeuro Pharmaceuticals, Rockville, MD USA

## Abstract

**Background:**

Alzheimer’s disease (AD) is a progressive neurodegenerative disorder that represents the most common cause of dementia in aged individuals. Given the central role of Aβ aggregation in AD, therapeutic strategies have focused on targeting Aβ to promote its clearance. Recent advances highlighted the need for antibodies with high selectivity for aggregated forms of Aβ, such as soluble Aβ (sAβ) aggregates and fibrils, while minimizing binding to monomeric Aβ. Monoclonal antibodies which selectively target aggregated forms of Aβ or amyloid aggregates which contain pyroglutamate modified Aβ have shown promising results in clinical trials, leading to their FDA approval despite the concerns for increased risks for vascular‐related side effects in certain groups of patients. However, there remains an ongoing need for more potent binding agents that selectively target aggregated forms of Aβ peptides and can be used for the diagnosis, prevention, and treatment of AD and other disorders characterized by Aβ aggregation.

**Method:**

Here, we describe the development of SNP234, a highly selective monoclonal antibody against sAβ aggregates that is more potent than currently available anti‐amyloid therapeutic antibodies. Its binding affinities to different species of Aβ were assessed using direct and competition binding assays. Human AD brain sections were used to assess its binding to endogenous, disease‐associated Aβ plaques. The antibody‐mediated Aβ clearance activity of SNP234 was also investigated.

**Result:**

SNP234 was one of several novel antibodies identified with high selectivity, exhibiting sub‐nanomolar affinity, for sAβ aggregates while maintaining low binding to monomer Aβ. It displayed excellent developability profiles amicable for subQ formulation. SNP234 binds robustly to disease‐associated amyloid and is efficacious in removing human amyloid plaques.

**Conclusion:**

SNP234 demonstrates high selectivity against toxic form of Aβ and promotes efficient removal of disease‐associated Aβ. These findings support further development of SNP234 as a potential next generation best‐in‐class anti‐amyloid immunotherapy agent with subcutaneous administration.

